# Reply to Dr. Barry's Remarks on the Gibraltar Epidemic of 1828

**Published:** 1831-04

**Authors:** Hugh Fraser

**Affiliations:** late Surgeon of the Civil Hospital, Gibraltar.


### GIBRALTAR EPIDEMIC.

*Reply to* Dr. Barry's *Remarks on the Gibraltar Epidemic of* 1828. By Hugh Fraser, Esq., late Surgeon of the Civil Hospital, Gibraltar.

(Continued from page 224.)

We have now to consider the case of my own servant-maid, commented upon at page 386 of Dr. Barry's Rejoinder. In my paper in the Medico-Chirurgical Review, I stated that this woman (Maria Piscadinha) was taken ill on the 3d of August, 1828: Dr. Barry thinks it is quite enough to set this point at rest, that he should declare, dogmatically, to the contrary: "I say," says Dr. B., "that she was attacked by this disease on the *twenty-third* of August." This statement the Doctor appears to have made, simply, because he finds the woman noted in Dr. Hennen's morning state of sick of the 2d and 3d of September, where, as I have already most distinctly explained, it was inserted by Dr. Hennen's order to Mr. Tucker, his clerk, at my suggestion, to enable Dr. H. to shew, in his official return for the month of August, all the cases of that particular form of fever which had come under my observation during that month; and that, in doing so, an error had been committed by Mr. Tucker, in not specifying the precise day of her having sickened; which error is by no means wonderful, considering the multifarious and very arduous duties which that gentleman had to perform at the period in question.

Dr. Barry has not attempted to explain why he omitted to bring forward the case of Mr. Hassan's servant, who had been attacked with the fever on the 17th of August, which was also noted, by Dr. Hennen's order, to Mr. Tucker, at my suggestion, in the same morning state; both patients having been, in this respect, similarly circumstanced. The bad faith, therefore, with which I have charged Dr. Barry, when speaking of these two individuals, has not, from any thing he has yet advanced, been removed.

Referring to a letter addressed by me to the British commission, dated on the 3d of January, 1829, relative to the early cases of the epidemic, and which Dr. Barry has brought forward, at page 387, for the purpose of proving that, because I did not state, in that letter, that my maid-servant had been attacked with yellow fever on the 3d of August, I had "either deceived the board of commissioners in 1829, or the public in 1830." The original draft of my letter to the secretary of the board, on the above occasion, stood thus:

"*Civil Hospital;* 3d *January,* 1829.

"Sir: In reply to your letter of the 28th instant, I beg to inform you that the first case of the epidemic fever which came under my observation took place in a child of Mr. Martin's, residing in No. 24 district, on the 18th of August, 1828. The history of this family is so generally known, that I deem it unnecessary to say more: *however, it may be proper to observe, that I have seen many cases of fever in the course of the year, which, to my view, bear a strong affinity to the cases I witnessed in the above family.* Should the board of commissioners require any further information from me, I shall be most happy to give it.

(Signed) "HUGH FRASER."

"To Dr. Barry,
"Secretary to the Board of Commissioners, &c."

At the time this was written, it was clearly my wish not to pass unnoticed the early cases of malignant fever which had occurred to me; but, on showing the above to my then colleague Mr. Wilson, he recommended me to omit the part printed in *italics;* as he admits, in a letter lately received from him, dated Cadiz, January the 19th, 1831, in which he says, "as it might be used as a controversial text, I recommended you to leave it out; upon which, you drew your pen or pencil through the paragraph, and there, I believe, the matter ended." The controversy to which Mr. Wilson refers, as my being then about to subject myself to, was regarding the word *identical,* in the circular letter of the secretary: all who should venture to pronounce that cases occurring before a certain time were identical with those occurring during the epidemic, being considered as men devoid of "*common sense!*" as was pronounced in a long article in an official journal published at Gibraltar, the credit of which was given to a certain person, under the especial sanction of others. My late colleague and very excellent friend thought at the time, that when so many subtleties and unfair means were adopted to disprove facts, when even black vomit was not allowed to be *the* black vomit, if not of the precise shade which some individuals choose to describe;* when yellowness was not *the* yellowness they were pleased to consider peculiar to the Bulam. In short, Mr. Wilson's observations led me to agree with him, at the time, that I was about to throw down the gauntlet, as it were, under great disadvantages; and, ultimately, with little benefit to science. I, therefore, like all others in the garrison, confined myself, in my letter, to the

* By the way, we have one source of cavilling annihilated, by Dr. Barry's admission, in his paper read to the College of Physicians, that the black vomit may be a "black brown."

period at which the first epidemic cases occurred, as generally acknowledged. That I adopted the measures, on this occasion, which a fuller consideration of the circumstances, perhaps, required, is what I shall not now pretend to say; but, with men of candour, it will be enough to show that I had not, as Dr. Barry would insinuate, been employing myself, unworthily, in framing cases for a particular purpose. This woman having been in my own service when attacked, and not admitted into hospital, was the cause of her case not having been registered in the books of the institution; that she was attacked with fever early in August, as I have stated; and that nausea, followed by yellowness and other marked symptoms, took place, could, if necessary, be established by the members of my family. That a discrepancy should have taken place between Mr. Wilson and myself respecting this woman, may be accounted for from that gentleman having been absent during the greater part of the month of August, as is stated by himself in the letter I have just mentioned to have lately received from him, and in which he says, "I left Gibraltar, for Cadiz, on the evening of the 5th of August, 1828, and returned towards the end of the same month: previous to my going, you had been two or three days in the country, and just returned as I started, or, at furthest, an hour or two before. I distinctly remember being in your house, to give you an account of what had gone on in the hospital during your absence, and I have a faint recollection of Maria being at the time unwell; but I cannot positively charge my memory as to what was done or recommended in her case." It appears, then, that Mr. Wilson, when speaking of this woman in his Historical Sketch, confounded her relapse on the 27th of August, with her primary attack on the 3d.* Mr. Wilson fixes the exact period of my return from the country, when I found that this woman had been ailing for two days previous. The superintendent and his secretary wished me, repeatedly, in private, to enter on explanations respecting this woman; but my invariable reply was, "Whatever you wish to be informed about, you must question me regarding it, officially, before the board." Strange to say, this was never done, although I had been frequently interrogated, upon minor matters, before that board. The two gentlemen just mentioned were aware, perhaps, that I might request a committee of medical men to inquire into the circumstances of

* Dr. Smith, in his paper in reply to Dr. Pym, remarks, "and on the 3d or 4th of August, Maria Piscadini, servant to Mr. Fraser, was attacked, recovered partially from this attack, and relapsed on the 27th of the same month."

this woman's case, as well as of others actually on my registers, previous to the arrival of the "suspected" ship Dygden, and of so decided a character as to place in their true light all efforts to prove the importation of the disease.

Dr. Barry, in his paper, read to the College of Physicians, states, (page 92,) when speaking of the two Fani children, already noticed as having died about the middle of August, 1828, "These are the very first cases of fevers that occurred on shore from the 14th of the preceding April, of which there is even a *tradition* discoverable." Now, really, we might well be excused for giving way to just indignation, when we observe that, on questions upon which so much depends, any body can have the hardihood to stand up, and make statements before a learned body so directly calculated to mislead. There would seem, indeed, to be no limit to the sacrifices which, in England, as well as in Gibraltar, this gentleman has made, in support of the cause to which he has given in his adhesion. My registers having been for some time in possession of the board of which Dr. Barry was the secretary, and that gentleman having had, besides, subsequently, access to them, as he admits, must have seen cases largely detailed for 1828, previous to the arrival of the "suspected" ship, which could have left no doubt as to their true character, in the mind of any man possessing the smallest knowledge of the disease. For myself, I must conclude, that Dr. Barry did recognize such cases as being registered in my books, not only for the year 1828, but in previous years; for, upon one occasion, in particular, I pointed out to Dr. Pym, publicly, at the table of the board of commissioners, and in the presence of Dr. Barry, a case so registered;* but the Doctor has endeavoured to conquer the embarrassment into which such

* The subject was Solomon Benshimol, admitted into hospital on the 3d of May, 1826, having been ill two days previous; and, as stated in the register, "countenance bloated," "body of a pale lemon colour," "copious coffee-coloured vomiting," "delirium" (more properly stupor), *death*. Upon this occasion the following dialogue occurred between Dr. Pym and myself. Mr. Fraser: "Here, sir, (pointing to the passages in my register) are the details of a genuine case of yellow fever." Dr. Pym, after glancing over the case, and, directing his words to the gentlemen of the commission, but more particularly to Mr. Judge Howell, "Poh! this is not a case of my fever, because the man did not die until the *tenth* day of his illness." Mr. F: "You perceive, sir, the man had black vomiting." Dr. P.: "No, no; you mistake black vomiting for the vomiting of coffee given to the patient before death." Mr. F: "Sir, the man does not appear to have used coffee." Dr. P.: "Yes, yes, yes, (closing up the register with vehemence,) oh yes, yes;" upon which, I took up my register, and left the board table.

cases would naturally place his friend and protector, by having recourse to a little of the management of which he was acknowledged to have been so capable. The Doctor, accordingly, in the sentence just quoted, speaks of the *14th of April,* by which he gets rid of two very remarkable cases which were treated in the Civil Hospital, and are registered in the books of that establishment: the one, Khian Bensimol, admitted so early as the *11th of January,* of the year 1828.* In this man there was not a single characteristic symptom of the Bulam absent: along with other symptoms of common occurrence, the suffused eyes, peculiar jactitation, marked redness of the margin of the tongue, suppression of urine, and, finally, what has been so much insisted on by Dr. Pym as particularly characteristic, the pale yellow or lemon colour of the skin; and most abundant discharges from the stomach of a fluid, as is expressed in the register, "black as mushroom juice," for two days before his death, which happened on the 16th. The second case is that of Solomon Anahony, admitted on the 13th of March, 1828: along with other symptoms, as gastric irritability, &c., this man had also the well-marked redness of the margin of the tongue; skin and conjunctivæ yellow, tongue vividly red and dry, as the disease advanced; collapse; countenance anxious and shrunk; mild delirium. This man recovered.

A third case occurred on the 23d of July; that is, after the period specified by Dr. Barry. The man's name was Macluf Benunez: previous to the attack, he seemed to have been labouring under a chronic visceral complaint: there was here, along with other grave acute symptoms, the symptom of

* The occurrence, occasionally, of *early* sporadic cases, some years, in Gibraltar, is very remarkable: since the epidemic of 1828, a case occurred in Private J. Musgrave of the 23d regiment, an officer's servant, residing out of barracks. This man was admitted into hospital on the 3d of March, 1830, having been ill for two days previous. He was seen, during his indisposition, by many of the medical gentlemen of the garrison; and the symptoms were of the most marked character; the morbid appearances were also of the most decisive nature, as witnessed by no fewer than *twelve* medical gentlemen who were present at the autopsy. A special report of this case was made to the director-general of the army medical department, by Dr. Smith, surgeon of the regiment, and may, I believe, be seen at the medical office in London. Various well-marked cases occurred at Gibraltar, in the autumn of the same year; and some of them also proved fatal. From whence did the ship come which introduced the contagion producing those cases, and why did not the disease spread? This will be answered by saying, "Oh, we don't admit those to be cases of the fever of which we speak!" The identity, however, is now put beyond the influence of all such cavilling, as any body who chooses to take the trouble may ascertain.

abundant vomiting of a fluid quite black, (stated in the register to resemble "*inky matter,*") considered so decisive of the true yellow fever, as also yellowness of the eyes and skin, singultus, and black stools. Death took place on the 26th.

A fourth case occurred, and this also subsequently to the period specified by Dr. Barry, in the person of the Jewish servant of the Civil Hospital, and which is also detailed in my register. The symptoms in this case, although not so well marked as in the three preceding, were still sufficiently so to have left no doubt, had the case occurred during the epidemic. The man was attacked on the 14th July, with nausea and distressing vomiting, which endured for two days; the skin became yellow, and he passed black stools. The two fatal cases having been Jews, no autopsies could be made.

As black vomit (considered by Dr. Pym so very characteristic of his "Bulam,") occurred in two of the above-mentioned cases, cavillers are deprived of their usual subterfuge when sporadic cases are mentioned, namely, "that such cases are of a very different kind, being always combined with inflammation of the liver." Aware of the importance to the cause of truth of its being duly established, by competent persons, whether the three cases (that of the Jewish orderly, owing to the comparative mildness of the symptoms, was not brought forward,) above mentioned, two of which we have seen to have occurred before the arrival of the "suspected" ship, corresponded in character to well-marked cases during the epidemic; aware also of the importance of having it established, once for all, by an examination of the registers of the Civil Hospital, whether sporadic cases of true yellow fever had, or had not, been treated, in previous years, in that establishment, not only by myself, but by other medical officers who had held the situation of surgeon to the institution, antecedent to my appointment in 1825, I solicited a meeting of several medical gentlemen of the garrison, and the following certificate was drawn up, after the cases had been read over from the registers: it will be seen that Dr. Chervin was present on the occasion.

"*Gibraltar; April* 13, 1829.

"We, the undersigned, have this day heard the foregoing thirty-nine cases, which have been extracted and condensed from the records of the Civil Hospital, carefully read over, and which have been compared with the original text. We are unanimously of opinion, that, with the exception of Case No. 23 (Samuel Bird), they are identical with the cases of the epidemic fever which existed in this garrison during the latter part of the year 1828.

(Signed) "John Gallice, Assistant Surgeon 12th Regt.
"George Brown, Assistant Surgeon 43d Regt.
"A. Brown, M.D. Assistant Surgeon 23d R. W. F.
"J. Miller, Staff Assistant Surgeon,
"Edw. Dow, Acting Deputy Inspector of Hospitals,
"T. Gillkrest, M.D. Surgeon 43d Regiment,
"Hugh Fraser, Surgeon of the Civil Hospital,
"R. Amiel, Surgeon 12th Regiment,
"—— Chervin, D.M.P."

All the gentlemen, in the above list of names, had seen yellow fever epidemically, several of them on more than one occasion; and it may be here proper to remark, that I made two official applications, at different times, to have the books and records of the Civil Hospital examined by a board of medical officers; but was unsuccessful on both occasions: I, therefore, of myself, requested a meeting of those gentlemen whose signatures we have just seen.

Now, as the above two cases of Rhian Bensimol and Solomon Anahony are seen to have occurred in 1828, long prior to the illness of my servant-maid on the 3d of August; prior to other similar attacks, which appeared at different and remote corners of the garrison,* particularly in the month of July, and prior to the illness of Mr. Hassan's servant; (the case of the latter we have seen, for reasons quite apparent, is altogether blinked by Dr. Barry;) I say, that a knowledge of these two cases, and including that of Macluf Benunez, being among the *thirty-eight* cases pronounced by the above gentlemen as being "identical with the cases of the epidemic," nothing can, I think, be more evident and conclusive, than that it was not necessary (were it possible that I should be so inclined,) that I should give the woman's case, as Dr. Barry insinuates, a colouring to answer a certain and most wicked purpose. The public will, I am sure, agree with the gentleman who had the honour of being secretary to so many boards, as to the importance of ascertaining *first cases* in epidemics: they have now, on evidence much less questionable, I will venture to affirm, than any in the power of that gentleman to produce, proofs sufficient to establish that, in Gibraltar, in 1828, as has so often happened in Spain in epidemic years, the occurrence of solitary cases, before the arrival of a suspected ship. This has, indeed, been so fully

* Vide Dr. Smith's paper.

proved, sometimes, with respect to Spain, that contagionists have been, not unfrequently, driven to shift *the blame* from a vessel first named, to another whose arrival was anterior, according as they found that their proofs of importation were overthrown. Had it been practicable to effect such a manœuvre with our epidemic, I need not say that any vessel, however remotely suspected, would have been eagerly grasped at by certain persons; but the truth is, as that independent officer, Colonel Chapman, so openly and boldly proclaimed, in his official opinion, as a member of the British commission, "after months of previous inquiry by those who wish to prove it," (importation,) no other vessel excepting the *suspected* ship Dygden could be found, upon which the "Bulam" gentlemen could affix their shattered and shipwrecked cause: on board of that ship, I think I may be permitted to remark, *en passant*, from what is now before the public regarding her, that there are no proofs whatever of yellow fever, or any other malignant disease, having existed, from the time she sailed from the Havannah, until the date of her receiving pratique in the bay of Gibraltar.

Following the secretary through all his mazes, we now arrive at the question of *second* attacks in the yellow fever; but here, I fear, we shall find the "impenetrable shield," of which Dr. Barry speaks in this part of his Rejoinder (p. 389), obscuring from his understanding not only facts resting on the clearest evidence, but the import of the very plainest language:" his being obliged to "guess" at what I meant by "moral testimony or evidence" confirming this or any similarly disputed question, seems to me rather extraordinary. I think, if he had consulted his dictionary, a better expression might have been hit upon for his Spanish confederates than the word "tradicion," which he tells us they adopted to get at what I meant to express by "moral testimony or evidence." I believe, I may venture to say that, in this country, moral evidence is considered as bearing the same relation to evidence of the most positive nature, as a moral certainty does to positive certainty. Thus, in reference to evidence before the commission appointed at Gibraltar, by the special desire of Dr. Pym, to inquire into the question of second attacks of yellow fever, nothing short of the *positive* evidence of black vomit in the first attack was required, before proof of a second attack would be admitted. I may be wrong in the application of the words I used, but I am as little disposed to acknowledge Dr. Barry to be a proper *censor* upon this, as I am upon any other question in which I am concerned; especially as I find him using the expressions "*lay inspectors*," "*lay*

*authority,"* in his Code Sanitaire; for I believe the application of the term *lay* to be always in opposition to *clergy*, while *civil* is used as opposed to *military*.

The question of non-liability to a second attack of this fever is a point which affects certain celebrated contagionists, in more ways than one: hence we find Dr. Barry dwelling on it in his Code Sanitaire for Gibraltar, as well as on every other possible occasion, and loading his friends with eulogies, *usque ad nauseam*, for what has been called a *modern discovery*.* But let Dr. Smith's paper be looked into, and it will be there seen that an alleged exemption from a second attack of yellow fever was publicly announced in placards on the corners of the streets in a town in Spain, some time before Dr. Pym laid claim to the discovery: let but old works on yellow fever be consulted, and it will be seen that observations, to the same effect, were made, *about eighty years ago*, in America: let the works of modern writers, French, Italian,† Spanish, and British, be consulted, and it will be found that, although it seems quite true that, on some occasions, the exemption from second attacks is very remarkable, on others this is not the case: see particularly, on this point, what Dr. Ferguson has stated as the result of his observations in the West Indies.‡ For myself, I agree, unhesitatingly, to the statement of second attacks not having been of *common* occurrence in Gibraltar; but I am in possession of a list of several well-authenticated cases, and others, I perceive, have been collected by the French commission, in their domiciliary visits, and noted in their documents lately printed.

On the point of insusceptibility to second attacks, Dr. Pym meets with the usual firm support from his late secretary; that is, if bold assertions be firm support; for, far from mincing matters, he roundly asserts, both in his paper read at the College of Physicians, and more recently in his Code Sanitaire for Gibraltar, which he appears to have furnished for the good of the present and future generation, that "this disease never attacks the same individual more than once." What conclusion is to be drawn when, in the face of such a statement, we have the signature of this gentleman to the occurrence of several cases of second attacks of yellow fever in Gibraltar and its territory? The gentlemen of the French commission made, as already stated, domiciliary

* "And, with respect to *my discovery* in yellow fever, I cannot give it up to the Spanish practitioners." (Dr. Pym, Edinburgh Medical and Surgical Journal, vol. xii. p. 135.)

† Among Italians, Dr. Arditi on the Cadiz Epidemic of 1800.

‡ Medico-Chirurgical Transactions, vol. viii. p. 9

visits, after the epidemic had ceased: they made strict inquiries, from the heads of many families, on this, as well as on other points; they received information of the occurrence of cases of second attacks; they made daily notes of the evidence which they collected, to which they affixed their signatures, and these notes are countersigned by the *ci-devant* secretary, who, as I have mentioned, was nominated by Dr. Pym, together with Mr. Peter Wilson,* to make visits with those gentlemen. The documents here referred to are now published by the French government, so that we have evidence, as good as need be, of second attacks of the Bulam at Gibraltar, and evidence, as good as need be, of the intrepidity of Dr. Barry. Circumstances such as these cannot, I presume, fail to have due influence on the minds of the public. To little purpose will it be hereafter said, "*these were not true cases.*" I say, then, what are to be considered true cases, when the evidence of a medical man cannot, on all occasions, be procured to substantiate the statement of individuals? One of the reasons† which has occasioned such ardour on the part of certain persons, for some years past, in supporting the non-liability to second attacks, is a desire to establish a family alliance between the Bulam fever and the exanthematous diseases, such as smallpox, measles, and scarlet fever; but what destroys all analogy between the "Bulam" and these diseases, is, as was very properly noticed by a gentleman at Gibraltar, that, in the yellow fever, *relapses*, which *never*, I believe, occur in the eruptive diseases, occur frequently in the "Bulam:" this, in a sweeping way, has also been denied; but, independently of numerous cases which fell under my own observation among the civilians of Gibraltar, I can bring forward the names of more than one hundred cases which occurred among the military, copied from the lists given into the Medical Office, and duly vouched by the signatures of the gentlemen in charge of hospitals.

With regard to the commission of which I speak, I admit it to have been composed as Dr. Barry states: that gentleman having had the honour of being named secretary, and Dr.

* We have seen the motives which induced my honourable friend and late colleague to withdraw himself from this commission; they cannot be too often repeated: "As I (Mr. Wilson) could not submit to the idea of putting my name to papers officially drawn up in this partial way, I thought it best to withdraw myself entirely."

† I speak of *one of the reasons* here, for others there are, report circulates, that a *pecuniary reward* was sought after for this "*discovery!*" Connected with this point, the College of Physicians was referred to some years ago, and declined pronouncing in favor of the *non-liability*.

Louis, one of the members of the French commission, president. To what I have stated in my paper in the Medico-Chirurgical Review respecting this commission, I have no disposition to add any thing more, although I could, without impropriety, very well do so: it is sufficient to repeat, that its proceedings were conducted upon principles so extremely objectionable as to induce both Dr. Chervin and myself to refuse to attach our names to them: indeed, as that eminent physician and philanthropist very properly observed, at the time, they seemed to be conducted with a view to impress a belief on this and future generations, that Dr. Pym had earned the laurels of a Harvey.

Dr. Barry asks, why the College of Physicians does not influence the government to print the proceedings of this report? I apprehend that this learned body know very well what they are about, when they refrain from recommending, and the government, I apprehend, knows what it is about also, in not undertaking the printing of the matter of this second-attack commission, or the matter of the British commission either, of which Dr. Barry was also the secretary. Perhaps a fuller answer may, one day, be given to the secretary's abrupt question on this point.

I now approach a part of the controversy which may be considered to be of high interest to the public, as the issue is calculated to throw great light on the question of contagion in the yellow fever. In my original paper in the Medico-Chirurgical Review, I stated that, "for the period of about one month from the commencement of the epidemic," hospital servants (soldier servants, or orderlies,) were not attacked; that, when cases occurred among them, it was not until the fever reigned among the inhabitants generally in the "*south,*" as it is called, (about one mile from the town,) being that part of the garrison where a large hospital for six or seven regiments, called Naval Hospital, is situated; and that, then, the first attacked were not those in close attendance on the sick of the different regiments, but cooks and watermen, whose duties did not oblige them to go near the sick, but who were, more commonly than other hospital servants, in the habit of sleeping on ground floors. Now, taking, for a moment, the commencement of the disease, in an epidemic form, from the period of its appearance in the Martin family, Silcocks, &c. (end of August:) but, to be more rigid regarding the military, let us take it from the 2d of September, the day on which the first soldier attacked (Sergeant John Holdroyd, 12th regiment,) was received within the walls of the hospital: what I expressed by "*a period of about one month*" ought, surely, in any thing like fairness, to be considered as from the end of August, or beginning of September, to somewhere about the 25th, 26th, or 28th of September. Let us now examine the shiftings which the exigency of his case obliges the secretary to employ upon this point: he tells the public that he has duly certified returns, proving that, from the "*5th of September to the* 10*th October (!)*" fifty-nine hospital attendants were attacked; *a period exceeding by not less than a fortnight that which I have stated;* a duration of time sufficient to account for the illness of double the number which he says were attacked, so potent did the poison of the disease become towards the end of September and beginning of October. Nobody, indeed, ever attempted to deny that, from the end of September, the permanent hospital attendants (like the people in the district generally in which the hospital is situated, and who attended no hospital,) fell ill rapidly. This appearance of the disease springing up at a later period in this part of the rock, has been observed in all the former epidemics of Gibraltar, and, in 1828, was foretold, with extreme precision, by some of the old residents of the garrison: the circulation of air in this locality, though very imperfect, is much superior to that in the town. To the statement I made as to no servant in attendance on the sick having been attacked for "the period of about a month," I cannot, after every information which I have since received from Gibraltar, find more than a single exception, namely, that of a man of the 94th regiment, attacked on the 6th of September: but, as in every other instance, whether civil or military, at the commencement of the epidemic, this individual had been, according to the statement of the medical officer in charge of the regiment, exposed to the *malaria* of the town; indeed, of the very district (No. 24) which was then the focus of the *infection.* The next hospital attendant, according to my information, fell ill on the 24th of September, but, agreeably to the medical officer of his corps, the case was one of *hæmoptysis,* not fever; and then, on the 25th, a cook of the 12th regiment, with a genuine fever;* next,

* Dr. Barry would give the public to understand (p. 390,) that the introduction of a Sergeant Holdroyd, (the first soldier in the garrison attacked with fever) into the hospital of the 12th regiment, on the 2d of September, kindled the spark of *contagion* throughout the whole of the other hospital establishments; but let us just hear what Mr. Amiel, the surgeon of the corps, says, in his official reply to queries from the Army Medical Board respecting this subject: "It is a well known fact, that the fever spread in the south two or three weeks later than in the town: from the 2d of September to the 1st of October, several cases of the epidemic were admitted into the regimental hospital, three of which died with the black vomit; but the *disease never attacked any of the other patients (more than twenty in number,) treated during the above*

a cook also, in the 43d regiment, on the 30th of September; then, in the same regiment, a waterman, on the 2d of October. After this period, the disease proceeded with a sweeping and an appalling energy; there was no safety in the western or sheltered side of the mountain; persons who had sought refuge, at the commencement of the fever, from the town, were now driven from their abodes at the south, in consequence of the disease breaking out around them in every direction, to the Bay, Neutral Ground, Europa Flats, and Windmill Hill; localities that remained perfectly free from disease, notwithstanding daily, nay hourly, intercourse was kept up between the town and those places, not only through persons, but articles, such as bedding, which might have been supposed to contain the very essence of contagious poison, or virus, had such ever existed. With respect to the servants of the Civil Hospital, I must refer to the details respecting them, already before the public, in Mr. Peter Wilson's Historical Sketch.

We have been talking of the permanent establishment only of hospital servants, in the military hospitals; but mark now how my expert antagonist has altogether blinked the statement which I made in my paper (p. 348,) respecting the *sixty-nine* men of one regiment (43d,) exposed, in daily parties of from three to six men, for twenty-four hours each, to all the various duties at the bedside of yellow-fever patients: let the result of such an experiment, if I may so call it, be referred to, and it will be seen that, at the end of the epidemic season, no greater number of those sixty-nine men, so exposed, in every possible way, were found to have had attacks, than those who never went near either sick persons or hospitals; and those of them subsequently attacked within three months had, as has, I believe, invariably been the case in all instances of persons attacked, been exposed to the

*period for other complaints,* nor *any of the orderlies,* who had as usual an incessant and unreserved intercourse with the dying, and slept in the same wards. It was only on the 25th of September, when the disease had spread in the south, and when the epidemic influence had extended to the district where the hospital is situated, that the cook of the establishment, who never had occasion to approach the sick, contracted the disease; and in the month of October, when the atmospheric causes had acquired more intensity, *the hospital sergeant, and twelve orderlies,* successively sent from the camp, were taken ill but a few days after entering the precincts of the hospital, and several of them fell victims to the disease." Again; "I have not been able, in any instance, to trace the progress of the disease from a single point; neither did I observe any subsequent attack in the company or regiment, that had any apparent connexion with the case of the sergeant (Holdroyd) whom I mentioned to have been the first sufferer." It is remarkable, as I stated in my paper in the Medico-Chirurgical Review, that this man (Holdroyd) was in the habit of visiting a *paramour* residing in the foul locality of No. 24 district.

deteriorated atmosphere of certain well-defined localities, but more especially the town. Dr. Barry has, as I have said, blinked the history of those sixty-nine men so fully exposed to contagion, had it existed; and, I believe, for a very good reason: I suspect the Doctor found himself, for once at least, fairly at fault, having been obliged to forward, to the colonial office, in his capacity of secretary, a list of the men's names, periods of duty at the hospital, guards mounted in town, &c., subsequently to the hospital services of the aforesaid men, all duly verified by the signature of the adjutant of the regiment to which they belonged. The number of men exposed, in the instance to which I refer, to whatever can be supposed to have been elaborated from the bodies of the sick on whom they attended, was so considerable, and the result so completely subversive of the doctrine of contagion, that I consider the document in question, of the most important and conclusive kind, so far as the history of yellow-fever contagion is concerned. Another important point respecting hospital servants, studiously left untouched by this zealous supporter of a bad cause, is my statement as to the admirable illustration of the question of non-contagion which we had at Gibraltar, in the fact of no hospital attendant whatever having been attacked in those establishments, placed out of the influence of the deteriorated atmosphere of the rock; that is, at Windmill-hill hospital, situated on a high and airy spot at the southern extremity of the rock, and at the temporary hospitals outside the walls, near the glacis, at the northern extremity, although several of those attendants were susceptible, in the sense of not having had previous attacks;* so that matters seemed, at Gibraltar, to have been fairly put to the test, in every possible way; and as to the results connected with the attendants on the sick of hospitals, and, I may also add, with respect to that of private families, they have shewn themselves singularly corroborative of what authors have recorded to have been witnessed, during black-vomit fever epidemics all over the world, more particularly in Spain, North America, and the West-Indian islands.† To what I have stated respecting the hospital

* In the 12th regiment four men of this description were in attendance, and remained unattacked.

† With respect to Spain and North America, the most ample details on this point may be found in the writings of Dr. Chervin; and, respecting the West Indies, I would particularly refer to the writings of Dr. William Ferguson, Inspector of Hospitals, and for some time at the head of the medical department of our Leeward Islands. What the celebrated Humboldt states he observed during his travels in yellow-fever countries, is quite irresistible as to the intransmissible nature of black-vomit fever, under every circumstance of exposure and situation. ("Political Essay on New Spain.")

servants, out of certain localities, not having been attacked, though in close attendance on epidemic cases, I would now add, that the same fact occurred regarding the medical men; for, while several gentlemen were attacked, (indeed, a comparatively few only escaped,) who were on duty at the military hospitals at that part of the rock called South, and while several others, as well as myself, were attacked in town, both of which situations, be it observed, are backed by a mass of rock of from 1200 to 1400 feet high, it so happened that, in two yellow-fever establishments, placed on sites open to a pure circulation of air, *not one* of the following gentlemen, who arrived from England at the latter end of 1828, was taken ill, although several of them were in close attendance on black-vomit cases at those places: Dr. Barry, then staff-surgeon;* Surgeon M'Load, 42d regiment; Dr. Galiani, assistant surgeon 43d regiment; Assistant-surgeon Davies, 18th regiment; Assistant-surgeon Dick, 12th regiment; Assistant-surgeons Bulteel and Burket, of the 94th regiment; Assistant-surgeons Bell, Fagg, and Moore, of the staff! Let these facts, which I venture to say will bear any examination, be compared with the manner in which Dr. Barry has dressed up the statement of the military-hospital attendants, and it will be at once seen whether he has or has not been misleading both the profession and the public on one of the most important questions in medicine.

(To be concluded in our next.) p. 399.

* It was reported at Gibraltar, that this gentleman made an exception, he having had, as was stated, an attack while living in camp on the Neutral Ground: it is, indeed, quite certain that he himself, in a letter written, for a very particular purpose, to Dr. Broadfoot (who was, for a short time, at the head of the medical department of Gibraltar, after the death of Dr. Hennen,) made use of some such words, or as, I think, I may say, exactly the words, (as subsequently read in public,) "Don't forget mentioning that I had the fever:" alluding to Dr. Broadfoot's intention of writing to England; but the cause of truth requires me to state that we have also Dr. Barry's own words in proof of the illness to which he refers having been of a somewhat different kind; his own words taken down at his bedside, by a friend of his, were; "I had a slight attack of *cholera morbus* last evening, but I am now much better, though in bed. I shall be out in the course of the day." The letter of which this is an extract is now before me, and is dated the 18th of November, 1828. There can be no mistaking this; it was dictated while matters were quite fresh in his memory. A further proof, if further could possibly be wanted, is, that the Doctor's name was not in the daily or monthly lists of persons attacked.

